# A historical perspective on HLA

**DOI:** 10.1093/immadv/ltad014

**Published:** 2023-08-17

**Authors:** Walter Bodmer

**Affiliations:** Weatherall Institute for Molecular Medicine, University of Oxford, Oxford, UK; Department of Oncology, University of Oxford, Oxford, UK

## Abstract

The discovery of the history of the HLA system is reviewed from the earliest attempts at cancer transfers between mice, through the discovery of the mouse H-2 system on mouse red blood cells, the discovery of HLA class II and II antigens by use of sera from multiparous women, to the resolution of the HLA and H-2 functions explained by the attachment of intra cellular peptides to the HLA antigen grooves on the cell surface. The study of the associations between HLA types and diseases forms the basis for the subsequent extensive study of the genetics of human complex disease and phenotypes by GWAS (Genome Wide Association Studies).

The story of the discovery of the human major histocompatibility system, HLA, starts with early experiments in which tumours from one mouse were transplanted into another mouse to see whether tumour growth would be stopped by the recipient’s immune system, as Ehrlich had suggested in 1909. It was, however, soon pointed out by Peyton Rous, discoverer of the eponymous virus, that normal tissue was also being rejected. No account had been taken of the genetic heterogeneity in the domesticated mouse strains being used for these experiments. CC Little realized the importance of this underlying genetic heterogeneity, and so, by several generations of brother/sister mating, produced inbred, isogenic, mouse strains, some of which, including C57/Bl, are still widely used. With his colleague Tyzzer, Little then showed that transplantation of tumours within the inbred strains overcame the inconsistencies of the earlier experiments [1].

Peter Gorer, a pathologist training in London to be a geneticist, took up the suggestion by his mentor, the distinguished geneticist JBS Haldane, to look for red cell blood groups in mice like those known in humans. He was then able to show that it was the differences between the inbred mouse strains with respect to the dominantly inherited variants of his genetic system, called H-2, that controlled the tumour rejection [2]. It was the discovery of H-2, the mouse major histocompatibility system, that foreshadowed the discovery of the HLA system, the human equivalent of H-2, and the demonstration of the importance of these major histocompatibility systems in the overall control of the immune response.

Peter Medawar, the noted immunologist and scientist who shared the Nobel Prize for his discovery of acquired immune tolerance, was an early pioneer of the study of skin transplantation. He showed, using the rabbit as a model for skin grafting, that it was leukocytes not red blood cells that accelerated the rejection of skin grafts between different animals and shared the presumed genetic features with skin that were recognized by the body’s immune system [3]. This seemed to argue against a role for the H-2 system found on mouse red blood cells by Gorer for histocompatibility. This difference between mice and rabbits was a bone of contention between Medawar and Gorer until resolved by experiments that showed the H-2 antigens in mice, and their HLA homologues in humans were on the majority of nucleated cells in both species.

The work of Gorer and Medawar clearly raised the challenge of finding similar systems to the mouse H-2 in humans. One obvious source of potential antibodies was the serum from individuals who had had multiple blood transfusions. Jean Dausset was the first to report in 1958 an attempt to define blood groups on leukocytes using such antisera. He described very tentatively a possible difference in a pattern of serum agglutination reactions using sera from many multiply transfused patients tested on just 20 donors. He called this pattern MAC. However, as might be expected from individuals stimulated to make antibodies from a wide variety of transfusion donors with different genetic backgrounds, the patterns of reaction were too complex for ready further identification.

This problem was solved by the simultaneous description, in 1958, by Rose Payne and Jon van Rood that multiparous woman, who had not had blood transfusions, often had antibodies in their serum that agglutinated lymphocytes from the father and offspring, as well as a proportion of randomly chosen donors. By analogy with Rhesus disease of the newborn, this implied that the women during pregnancy had produced antibodies against lymphocyte surface determinants that their children had inherited from their fathers. These would necessarily be less complex than the sera from multiply transfused unrelated patients, which had been studied by Dausset. Both van Rood and Payne realised that such sera could be the basis for the detection of new antigenic systems on lymphocytes different from the well-known red cell blood groups that did not explain incompatibility differences between unrelated individuals.

Van Rood *et al*. using a computer, which was then quite a novelty, developed a simple 2 × 2 cluster analysis of the agglutination reactions of a panel of antisera from multiparous women on a random panel of leukocyte donors. The argument was that clusters of sera tending to react together would be the basis for the definition of an antigen. On this basis, they defined two antigens 4a (later HLA-B*w4) and 4b (later HLA-B*w6) controlled by two alleles at a single locus [4].

As a statistician and geneticist, I was very interested in unravelling the complexity of this emerging system of human leukocyte antigens and so with serologist Rose Payne, and with help from my wife, statistician Julia Bodmer, we formalized and extended the 2 × 2 cluster analysis to include negative correlations and applied this to a body of data similar to van Rood’s using a slightly different agglutination technique. The results were immediately striking in defining three clear-cut clusters of sera such that all the sera within a cluster tended to react similarly to the cells from a panel of different individuals. One of these clusters turned out to identify van Rood’s 4b. The other two clusters defined two corresponding antigens, which we called LA1 and LA2 (now known as HLA-A*01 and HLA-A*02), controlled by alleles at a new locus different from that controlling van Rood’s 4a and 4b alleles. Membership of a serum in a cluster was defined by a significant chi-square for a positive 2 × 2 association with all (or nearly all) the other members of the cluster. Associations between individual serum members, one from the LA1 and the other from LA2 cluster, were nearly all significantly negative, exactly as would be expected if the corresponding genetic variants for the LA1 and LA2 antigens were controlled by alleles at a single locus. The fact that there were individuals who had neither the LA1 nor the LA2 antigens showed that there must be other alleles, with their corresponding antigens, still to be found. That defined what is now the *HLA-A* locus [5]. These two studies, which identified what we now call the *HLA-A* and *HLA-B* loci, set the pattern for the whole future definition of the HLA system.

While Dausset’s MAC, loosely related to HLA-A*2, led him to share the Nobel Prize with George Snell, who largely developed the mouse-inbred strains and their H-2 types as we now know them, it was really Jon van Rood who set the scene for the discovery of the HLA system. No doubt if Gorer, the discoverer of H-2, had not died at an early age from lung cancer due to smoking he would also have been in line for a share of the Nobel Prize.

The further development of the HLA system was hugely stimulated by the initiation of the international HLA workshops. These were started by Bernard Amos, an early H-2 and HLA pioneer, in 1964 just at the time that HLA-A*1 and *2* were defined. The aim was to enable active working collaborations between different laboratories to agree on the definition of new antigens, and so loci and their alleles. Initially, this was done by each participating laboratory testing a common set of cells from a panel of donors with their own panel of antisera to see whether the results matched up with each other. These workshops gradually grew in their scope of activities and the widening range of participating laboratories from all over the world. They also, through an associated nomenclature committee, ensured a common language to define the HLA system and its variants as the eventually hugely complex system developed. This has been a remarkable example of international collaboration that has continued right up to the present with the most recent workshop, the 21st International Histocompatibility Testing workshop, taking place in Amsterdam in 2021.

Once the HLA system had been identified, originally by two loci and a few alleles, the obvious question was whether it really was the major genetic system that controlled histocompatibility, namely graft rejection. Van Rood first demonstrated a possible relationship to graft rejection by injecting leucocytes from a donor who differed by just one antigen from the recipient. He observed an accelerated rejection by the recipient of a skin graft from the donor who expressed this antigen, but no accelerated rejection of a graft from a donor who did not express the antigen. Such an experiment, which was reported at the second International Histocompatibility Testing workshop, organized by van Rood in 1965, probably would not be allowed nowadays.

The most striking early relationship between HLA matching and graft survival was shown by data obtained by Paul Terasaki *et al*. showing that kidney transplants exchanged between HLA identical sibs had enormously better survival times than those exchanged between non-HLA identical sibs. This worked because even the very early serological typing had suggested close linkage between whatever genes determined the types, so that on average 1/4 of sib pairs would be expected to be HLA identical even when so identified by comparatively few sera [6]. Their studies had been greatly enhanced by Terasaki’s invention of a microcytotoxicity assay, which soon, in various forms, became the standard assay for serological determination of HLA types.

The mixed lymphocyte culture reaction, in which lymphocytes from unrelated individuals stimulate each other to divide, was discovered independently by Bach and Bain in 1964. This stimulation was then shown by Bach and Amos in 1967 to be associated with HLA antigens in families by finding no reaction between HLA identical sibs within a family. This finding led to a serological search for the presumed associated surface determinants that caused this inter-lymphocyte interaction. Several groups of investigators found that these putative determinants could be identified by some reactions of sera from multiparous women that occurred with just the isolated B lymphocytes in the blood of a donor being tested rather than with all the white cells in the donor. This eventually identified a new class of HLA-linked surface determinants, now known as the HLA Class II DR, DP, and DQ antigens with related *HLA-DRB*, *DQA*, *QDB*, *DPA*, and *DPB* genes. These were first clearly defined at the 7th International Histocompatibility Testing workshop in Oxford in 1977. This then completed the major identification of the genes of the HLA system as we now know it.

The high level of polymorphism of the HLA system was already clear from the fact that highly polyspecific sera were produced by individuals who had received multiple blood transfusions and from the high frequency with which foeto-maternal stimulation produced HLA antibodies, this being the main source of sera used for the initial definition of the system. The early International Histocompatibility Testing workshops established that the major polymorphic determinants were controlled by multiple alleles at the two closely linked loci, HLA-A and -B, and then identified a third, even more closely related and linked locus, HLA-C. The definition of the Class II loci greatly expanded the overall level of variation generated by the HLA system, which helped to define patterns of variation between different human groups in different parts of the world that are further studied at each of the International Histocompatibility Testing workshops.

Following the cloning of all the genes in the HLA region, HLA typing is nearly always done now using DNA-based techniques. There are now thousands of alleles defined for both the HLA Class I and II genes, with rather more in Class I than II. Though there is substantial variation between different population groups in the patterns of frequencies of the more frequent HLA alleles, most of the alleles, especially those found more recently, occur at very low frequencies. There is, inevitably a huge range of up to 8 linked locus haplotypes, a term coined by the human geneticist, Ceppellini, another of the HLA pioneers. Certain haplotypes involving the six major loci and the more common alleles do, however, occur with measurable frequencies in one or more populations.

An excellent history of the HLA system up to 1990 was assembled by Terasaki based on contributions from all the major HLA pioneers ([7], and see also [8] for further references).

How does the HLA system function, what is its role in the immune system, and what is the practical medical impact of HLA variation? I will cover these questions only briefly as the answers depend on different perspectives.

The first clue to the functional role of H-2 types, and so of HLA types, was dramatically elucidated in a two-page paper by Zinkernagel and Doherty published in *Nature* in 1974 [9], for which they received a Nobel prize. They showed that T cells from a mouse immunized by the lymphocytic choriomeningitis virus (LCMV) would only kill LCMV-infected cells that were from mice with the same H-2 type. They concluded that this meant that immune recognition by the T cells depended both on the virus type and on the H-2 type of the attacking T cells.

It was then shown by Alain Townsend *et al*. in Oxford that peptide fragments from degraded proteins inside cells were presented on the cell surface by attachment to the H-2 Class I products [10]. This was the ultimate explanation of Zinkernagel and Doherty’s demonstration that T-cell recognition of immune targets depended on a combination of the target and the T cells’ H-2 type. This model for presentation of internally derived peptides on the cell surface also applies to the HLA Class II products.

In 1987, Pamela Bjorkman *et al*. at Harvard University elucidated the crystal structure of HLA-A*2, which dramatically showed how the peptides found by Alain Townsend bound into the groove of HLA-A*2 and provided a general model for peptide binding to all the different HLA gene products [11].

The possibility that there might be HLA associations with certain diseases was realized soon after HLA was first discovered. Striking associations of specific HLA types were found with, for example, ankylosing spondylitis and type 1 diabetes. These associations could be explained by applying the association between genetically controlled immune responses and H-2 types in the mouse, found by McDevitt *et al*., to the human situation [12]. The explanation of how such associations could be due to linkage disequilibrium with a variant at a closely linked locus [13] has formed the basis for genome-wide disease association studies.

There is really no aspect of understanding the immune system and its role in diseases, their treatment, and prevention, for example by vaccination, that does not to some extent depend on our knowledge of the HLA system. Some examples of future developments in cancer immunotherapy are discussed in [14], and the early important contribution of the development of monoclonal antibodies to HLA gene products to the study of HLA function is discussed in [15].

In this perspective, I have aimed more at the earlier developments of the HLA system, which may be less familiar to the current active generation of immunologists than more recent developments, and focussed mainly on references that deal with key developments of our knowledge of the HLA and other major histocompatibility systems.
